# Accurate Determination of Conformational Transitions in Oligomeric Membrane Proteins

**DOI:** 10.1038/srep23063

**Published:** 2016-03-15

**Authors:** Máximo Sanz-Hernández, Vitaly V. Vostrikov, Gianluigi Veglia, Alfonso De Simone

**Affiliations:** 1 Department of Life Sciences, Imperial College London, South Kensington, London, SW7 2AZ, UK; 2 Department of Biochemistry, Molecular Biology and Biophysics, University of Minnesota, Minneapolis, MN 55455, USA.; 3 Department of Chemistry, University of Minnesota, Minneapolis, MN 55455, USA

## Abstract

The structural dynamics governing collective motions in oligomeric membrane proteins play key roles in vital biomolecular processes at cellular membranes. In this study, we present a structural refinement approach that combines solid-state NMR experiments and molecular simulations to accurately describe concerted conformational transitions identifying the overall structural, dynamical, and topological states of oligomeric membrane proteins. The accuracy of the structural ensembles generated with this method is shown to reach the statistical error limit, and is further demonstrated by correctly reproducing orthogonal NMR data. We demonstrate the accuracy of this approach by characterising the pentameric state of phospholamban, a key player in the regulation of calcium uptake in the sarcoplasmic reticulum, and by probing its dynamical activation upon phosphorylation. Our results underline the importance of using an ensemble approach to characterise the conformational transitions that are often responsible for the biological function of oligomeric membrane protein states.

Membrane proteins (MPs) are fundamental biomacromolecules playing key roles in many cellular mechanisms and that are targeted by approximately 60% of the commercialised drugs^1^. Many MPs are homo- or hetero-oligomers^2^,^3^,^4^ and require a quaternary structure to achieve biological function. Oligomeric MPs are crucial molecules for processes such as ion channelling^5^,^6^, active transport through the membrane^7^, signal transduction^8^, storage^9^ and photosynthesis^10^,^11^. It is generally established that in order to elicit their biomolecular functions oligomeric MPs exploit collective motions of the backbone and side-chain atoms^12^,^13^,^14^,^15^,^16^, which also govern conformational equilibria such as self-assembly/disassembly or order-disorder transitions. One such case involves phospholamban (PLN), a 52-residue MP that exists in equilibrium between different forms, including structured (T-state) and partially disordered (R-state) conformations, monomeric/pentameric forms and states that are bound/unbound with the sarco(endo)plasmic reticulum Ca^2+^-ATPase (SERCA). The populations of these states are redistributed upon PLN phosphorylation at serine 16, a process that regulates calcium uptake in the sarcoplasmic reticulum by SERCA^17^,^18^,^19^,^20^,^21^. The metamorphic nature of PLN makes this system essentially intractable for classical structural techniques that are primarily tailored for the structure elucidation of well-defined and relatively rigid proteins. Indeed previous NMR structural refinements of PLN in micelles have been successful to refine the conformation of the structured T-state, only^22^.

In the present study, we present an optimal approach to characterise in detail the structure and dynamics of oligomeric membrane proteins by using oriented solid-state nuclear magnetic resonance (ssNMR) experiments in combination with restrained ensemble-averaged molecular dynamics (MD) simulations^23^,^24^,^25^. When treating experimental data of oligomeric MPs, the complex averaging of the NMR observables poses significant challenges. We show that ensemble-average MD can be used to successfully characterise structure and dynamics of the pentameric state of PLN in both phosphorylated (pS16) and non-phosphorylated forms. The structural ensembles resulting from this study describe accurately the nature of the dynamic activation of the pentameric PLN upon phosphorylation, which is an underlying mechanism of SERCA regulation.

## Results

### Structure and dynamics of oligomeric MPs by oriented ssNMR

**Figure 1:**
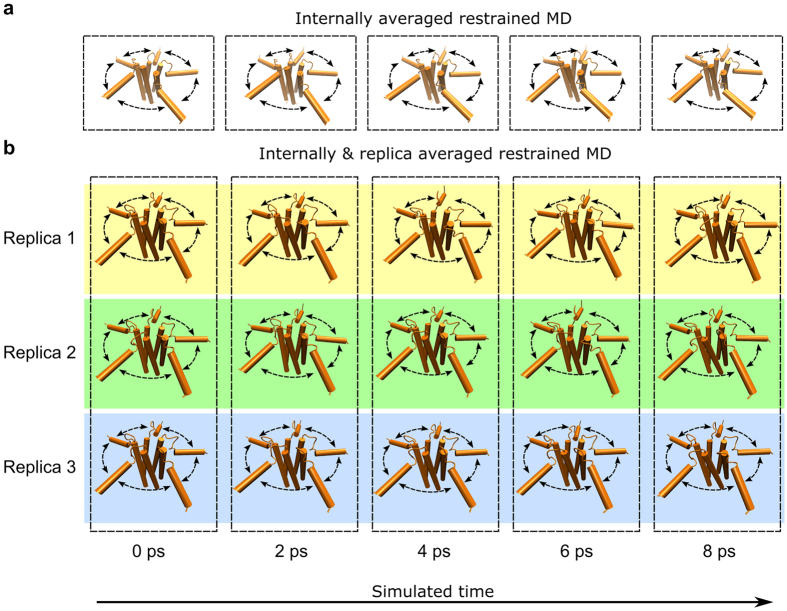
Internal averaging and replica averaging schemes. (**a**) Single-replica MD simulation restrained using the internal averaging, which implies oriented ssNMR data being averaged amongst the protomers of an oligomeric MP. (**b**) Internal averaging is coupled to replica averaging. In the example, three replicas are run in parallel. At every step, ssNMR observables are back-calculated from the atomic coordinates in all systems and their values are averaged across the replicas in addition to the internal averaging amongst the individual protomers of each oligomer. The back-calculated values are compared against their experimental counterparts to calculate the restraining energy term that is combined with the force field to drive the sampling toward an accurate characterisation of the structure and dynamics of the system.

Our study aimed at establishing a method to refine structural ensembles that could describe in detail the biological properties of oligomeric MPs, including those systems having a significant dynamical behaviour such as the pentameric state of PLN. To this end, we implemented oriented ssNMR data as experimental restraints in structural refinements exploiting ensemble-averaged molecular dynamics (MD) simulations^26^. In oligomeric MPs, NMR observables are averaged across the oligomers as well as the individual protomers composing each oligomer. To account for this property, we optimised restrained simulations that utilise an ensemble averaging scheme combining both “internal” and “replica” averaging, which average the calculated NMR data across individual protomers in the oligomers and across multiples copies of the same system (Fig. 1). These restraints were implemented by modifying the force fields employed in MD simulations with an energy term that depends on both atomic coordinates and experimental data (equation1).


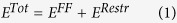


Where 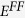
is the standard MD force field and 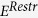
is the experimental restraint that is averaged across M replicas and P protomers (eq. 2).


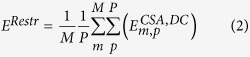


Where 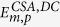 are the harmonic restraints of chemical shift anisotropy (CSA) and dipolar couplings (DC) in each replica (see Methods section).

The optimisation of this method included a calibration procedure defining the optimal number of replicas, the force constants by which the restraints should be applied and the proper combination of internal and replica averaging. This calibration was performed thorough the ‘reference ensemble’, which has been extensively described in the literature^27^,^28^ (Supplementary Material). This *in silico* experiment identified the optimal averaging scheme to be composed of 16 replicas of the system in combination with internal averaging (Fig. 1). We demonstrate that this approach can generate structural ensembles with an error reaching the statistical limit (Supplementary Methods and Fig. S1), which provides an estimate of the significant accuracy by which CSA and DC restrained MD are able to refine structure, dynamics and topology of oligomeric MPs.

### Refinement of pentameric PLN in lipid bilayer using experimental CSA and DC

**Figure 2:**
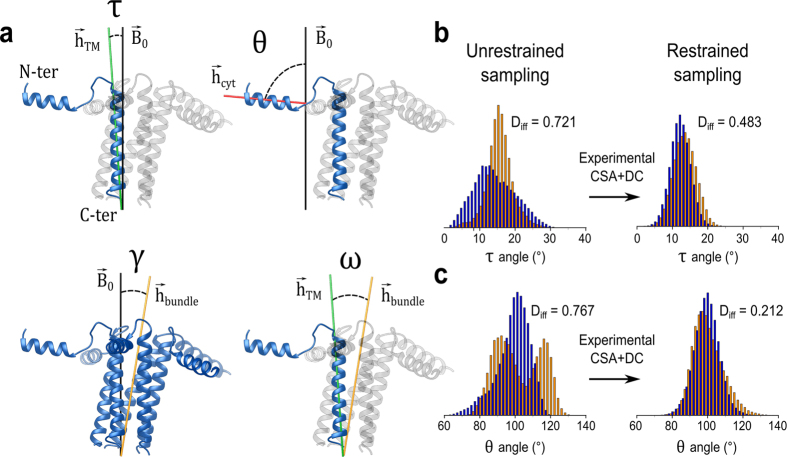
Topological properties of the phospholamban pentamer. (**a**) Tilt angles employed to characterise the topological properties of the PLN pentamer. The transmembrane tilt (τ), cytoplasmic tilt (θ) and global tilt of the helical bundle (γ) are defined between different axes of the pentamer and the external magnetic field, **B**_**0**_, whereas the ω angle is calculated in the internal protein frame and corresponds to the angle between individual transmembrane helices and the global axis of the transmembrane helical bundle. (**b,c**) Distributions of τ (**b**) and θ (**c**) angles in CHARMM-based (orange) and AMBER-based (blue) ensembles. Restrained ensembles (right) converge toward highly consistent topological parameters, as described by lower values of D_iff_ (see Supplementary Methods) with respect to the unrestrained samplings.

Using the identified optimal setup for refining structural ensembles of oligomeric MPs, we characterised the conformational properties of the pentameric PLN in explicit DOPC:DOPE lipid bilayer by implementing CSA and DC data, previously measured via mechanically aligned membrane systems^29^, as restraints in the ensemble averaged MD simulations. Our strategy to assess accuracy and convergence of these experimentally restrained simulations includes the production of two independent ensembles generated by restraining two different force fields, namely CHARMM36 and AMBER99SB-ILDN. When employed in standard MD of the pentameric PLN, the original force fields generate ensembles showing considerably different structural and dynamical properties (Fig. 2b,c). In contrast, the ensemble-averaged MD simulations, extended up to 100 ns for each of the 16 replicas, converged toward conformations showing a significant agreement with experimental data (Fig. S2), with Q factors of 0.035 (0.037) and 0.095 (0.099) and standard deviations of 8.62 (7.76) ppm and 0.99 (0.90) kHz for CSA and DC, respectively (bracket values correspond to the AMBER based restrained ensemble).

**Figure 3:**
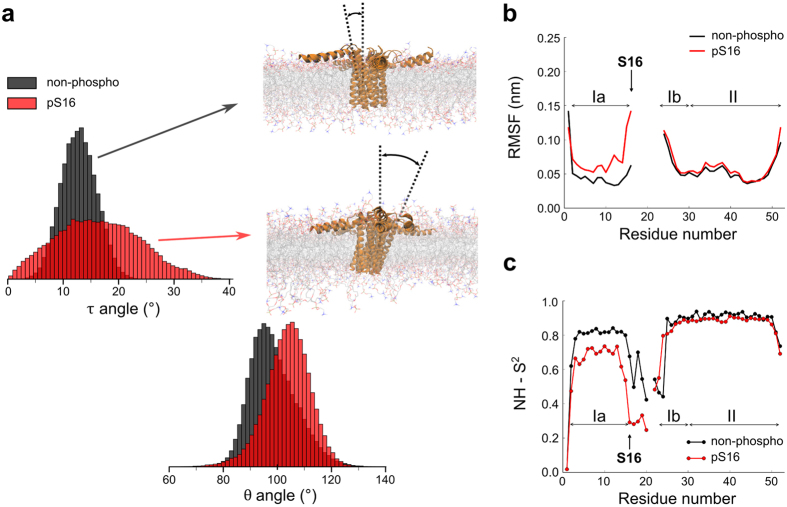
Dynamical activation of PLN pentamer upon phosphorylation. (**a**) Comparison of topological properties (τ and θ angles) of the PLN pentamer in its non-phosphorylated (black) and pS16 (red) forms. The transmembrane helices from domain II in pS16 PLN feature a wide range of orientations compared to the non-phosphorylated form. (**b**) Root mean square fluctuations (RMSF) for the two ensembles, calculated on the C_α_ atoms. The calculation was performed independently for domains I and II in order to remove biases from inter-domain motions. (**c**) Back-calculated order parameters (S^2^) of the amide nitrogen. This figure reports data from the restrained CHARMM36 sampling. These data are significantly convergent with those calculated from the AMBER99SB-ILDN restrained ensemble (Fig. S4 a–c).

Remarkably, despite using force fields having significantly different characteristics, the two restrained ensembles showed highly converging topological and structural features (Fig. 2b,c), suggesting that the experimental restraints in this method overcome intrinsic biases of the individual force fields. In these ensembles, the pentameric PLN preserved a pinwheel topology, with the bundle of transmembrane helices (domain II) firmly inserted in the membrane and cytoplasmic domains Ia and Ib adopting a parallel orientation with respect to the membrane surface. In particular, the distribution of τ angles (defined in Fig. 2a) in domain II is centred around an average value of 12.8° ± 3.17, in excellent agreement with the angle estimated using a static model to fit the PISEMA data^30^ (Fig. S3a). Conversely, the cytoplasmic region (domain Ia), which in both ensembles is adsorbed on the membrane surface showed a significant topological variability, with θ angles (defined in Fig. 2a) distributed at 98.8° ± 8.58, a larger root mean square fluctuation (RMSF, Fig. 3b and S4a), and lower values of the order parameters S^2^ than in domain II (Fig. 3c and S4b).

### Dynamical activation upon PLN phosphorylation

Phosphorylation of Ser 16 of PLN is a key post-translational modification to regulate the cycles of cardiac muscle relaxation by enhancing the population of R states in PLN^31^. We here address the effects of phosphorylation on the structural fluctuations of the pentameric state of PLN by producing accurate structural ensembles of pS16-PLN pentamer using CSA and DC measured in aligned samples^32^. The resulting experimentally restrained MD ensembles showed highly converging structural properties (Fig. S5) in excellent agreement with experimental data (Q factors of 0.033 (0.027) and 0.170 (0.173) for CSA and DC, respectively). These structural ensembles provided evidence of a conformational transition of the pentamer upon phosphorylation to assume increased dynamics in both domains I and II. This transition is in agreement with the considerable shifts of the experimental CSA and DC toward isotropic values (Fig. S6). In the transmembrane region, this activation results in domain II helices assuming a wide range of τ angles (Fig. 3a). Despite the larger topological variability with respect to the lipid bilayer, the quaternary interfaces of domain II are preserved upon phosphorylation, including the packing and mutual orientation of the transmembrane helices in the five protomers (Fig. S7). Thus, the ensembles provide a structural description for the isotropic scaling of CSA and DC, which includes enhanced dynamics in the helical bundle that preserve the quaternary structure at the protomers interfaces. In contrast, a static model to fit the PISEMA data^30^ gives rise to a τ angle of 20° for the helices of domain II that would effectively disrupt the quaternary interface held together by a Leucine/Isoleucine zipper, which has a limited tolerance for structural variations in the PLN pentamer^32^. While phosphorylation results in larger distributions of orientations in the transmembrane bundle, no alterations in the backbone nanoseconds dynamics are found, with back-calculated order parameters in domain II being consistent across non-phosphorylated and pS16 forms of the PLN pentamer, in agreement with previous ^15^N relaxation experiments^32^ (Fig. 3c and S4b).

A different scenario is found when analysing the dynamical behaviour of the cytoplasmic regions, whose nanosecond backbone dynamics are enhanced upon phosphorylation as shown by back-calculated order parameters S^2^ (Fig. 3c and S4b) and root mean square fluctuations (Fig. 3b and S4a) in agreement with relaxation data^32^. Domain Ia also shows differences in the topological properties, with higher values of the θ angle in the pS16-PLN pentamer than in the non-phosphorylated form (98.8° and 104.0° for the non-phosphorylated and pS16-PLN, respectively). These structural and dynamical properties are convergent among restrained ensembles calculated based on CHARMM36 and AMBER99SB-ILDN force fields (Fig. S5). Note that when used in unrestrained MD, these force fields give rise to non-convergent results in stark contrast with the experimental relaxation data^32^ (Fig. S8). This is an orthogonal demonstration of the accuracy of the structural dynamics described using restrained MD, showing that the method overcomes the intrinsic biases of the force fields employed to study membrane proteins.

### Channelling properties of the PLN pentamers

**Figure 4:**
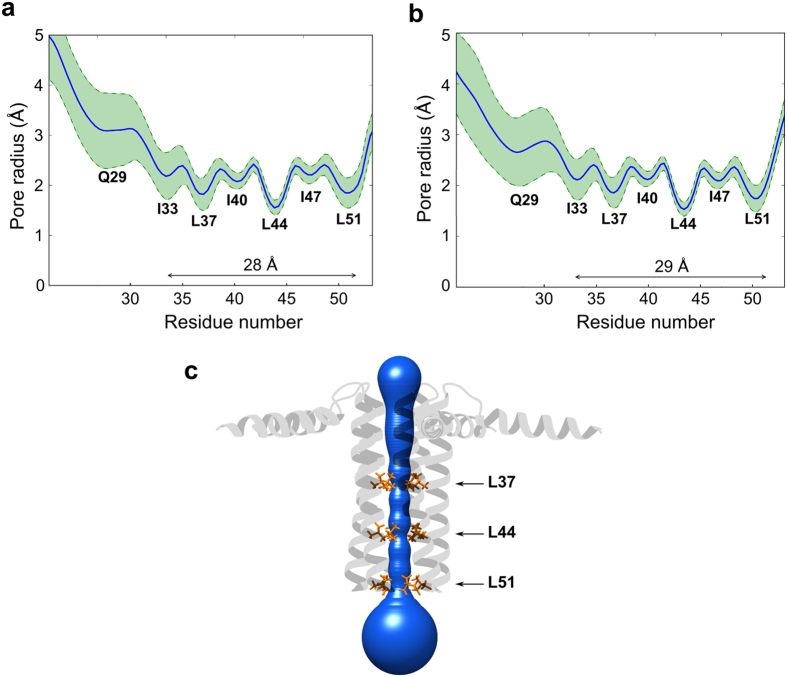
Characteristics of the interfacial transmembrane pore. The pore radii of pentameric PLN in its non-phosphorylated (**a**) and pS16 forms (**b**) have been characterised by using the program MOLE 2.0^56^. The data shown in this figure are from the CHARMM36 ensemble and are entirely consistent with the AMBER99SB-ILDN case. The mean value of the pore radius at the Z level of each residue is represented as a solid blue line. Green dashed lines represent the standard deviation of the pore dimensions in the ensemble. Each bottleneck is assigned to the corresponding residue facing the pore. (**c**) The average pore of the ensemble overlaid onto the PLN non-phosphorylated structure, with the three major bottlenecks shown in orange.

A central debate on the function of PLN is the putative ability of the pentameric assembly to favour direct calcium channelling through the pore formed at the quaternary interface between the transmembrane helices^33^. Recent structural characterisations and theoretical calculations, however, strongly dismissed this hypothesis by evidencing a tightly packed hydrophobic core at the interface between protomers that would make the Ca^2+^ transport energetically unfavourable^29^,^32^,^34^. In analysing our ensembles, we found that the pore retains three major bottlenecks that are positioned at the height of residues 37, 44 and 51 (respective radii 1.78, 1.54 and 1.81 Å), and shows an overall average radius of the 2.11 Å (Fig. 4a). In the case of pS16-PLN, the ensembles provided no evidence of alterations of the quaternary interfacial pore, with an overall average pore radius of 2.09 Å and all the three major bottlenecks preserved (Fig. 4b).

**Figure 5:**
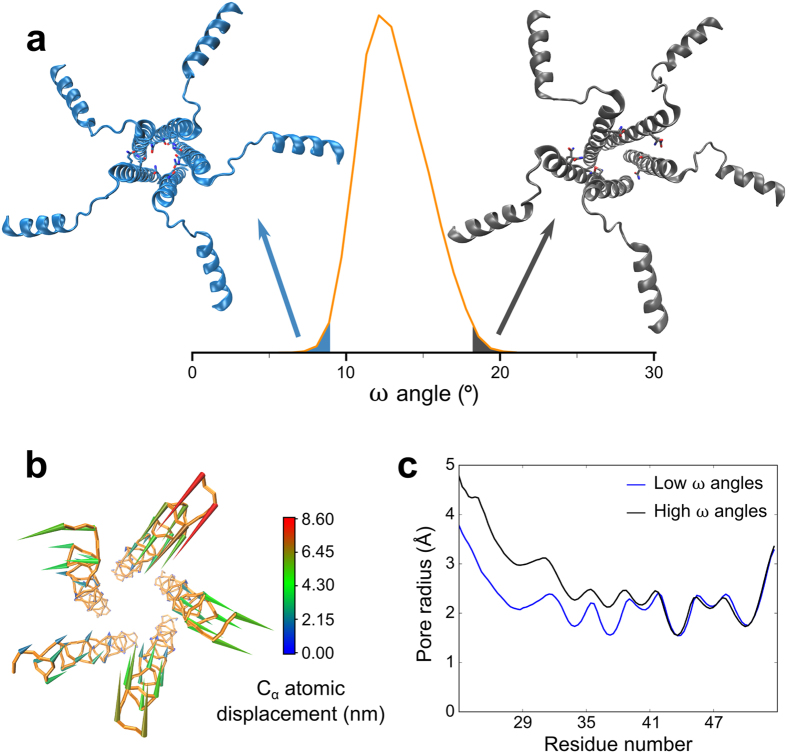
Internal motions of the transmembrane segment of pS16 PLN. (**a**) Distribution of the ω angle in pS16-PLN pentamer. The distribution is entirely conserved in the non-phosphorylated form (Fig. S5). Low values (blue) correspond to a highly symmetrical arrangement of helices in a circular shape, whereas high values (black) correspond to conformations featuring a more distorted and elliptical arrangement. Q26 and Q29 side-chains (shown as sticks) establish transient inter-protomer interactions that form at low values of the ω angle and are disrupted at higher values. (**b**) Porcupine plot showing the backbone motion mode of the transmembrane segments along the first eigenvector. Cones show the atomic displacement of C_α_ atoms, ranging from blue (small) to red (large). This diagram was produced using a tcl script available in the VMD^57^ program. (**c**) Differences in the pore radius profile between structures in the bottom (blue) and top (black) one-percentile intervals of the distribution in (**a**). Calculations were carried out in the CHARMM36 pS16 ensemble.

In addition to the average properties, principal component analysis was performed in the domain II to assess whether transient conformations can form along large-scale fluctuations in such a way to modify the channelling properties of the pentamer. The analysis described a collective motion in the N-terminal region of the domain II of the pentamer that results in the twisting of the five transmembrane helices assembly, particularly in the region spanning residues 25 to 40. This collective motion, which is conserved in both pentameric pS16-PLN (Fig. 5) and non-phosphorylated PLN (Fig. S7) states, is associated to the formation/disruption of intermolecular interactions between the side-chains of Q26 and Q29, which stabilise the quaternary interface at low values of the ω angle^35^ (Fig. 5a). At high values of the ω angle, this global twisting motion disrupts these stabilising interactions, resulting in loose local quaternary packing in the region spanning residues 25 to 35 but with no effects in the C-terminal part of the pore, which preserves the bottlenecks at positions 44 and 51 (Fig. 5c). As a result this analysis suggests that, despite the PLN pentamer exhibiting an increased dynamical behaviour upon phosphorylation, its ensemble does not feature any transient conformation that may favour the transport of calcium ions at the quaternary interface of the PLN pentamer.

## Discussion

It is crucially important to characterise the conformational transitions in oligomeric membrane proteins, as these proteins often exert their biological function via collective motions that are actively employed in processes such as signal transduction or molecular transport across the membrane. We devised an approach to refine structural ensembles of oligomeric MPs that can accurately describe these conformational transitions by combining two techniques that collectively probe both topological properties (oriented ssNMR^36^) and atomic fluctuations (ensemble-averaged restrained MD simulations^28^,^37^,^38^) of membrane proteins. Numerous studies have shown that the use of experimental restraints in MD simulations provides a powerful approach to sample conformational space of proteins by overcoming the intrinsic biases of force fields and limitations in accessible timescales^28^,^37^,^38^,^39^,^40^,^41^, which is particularly crucial when studying oligomeric membrane proteins, as these biases may significantly affect the topological properties of these proteins in MD^42^. This combination has been here applied by using two layers of ensemble averaging of the experimental restraints, namely replica and internal averaging (Fig. 1).

Using this approach, we could characterise the conformational dynamics in the pentameric states of non-phosphorylated-PLN and pS16-PLN. Our ensembles provide an accurate description of both slow (millisecond) and fast (nanosecond) internal motions in the pentamer and elucidate the structural bases of the enhancement of the pentamer dynamics upon phosphorylation. In particular, phosphorylation enhances the millisecond dynamics, as sampled by CSA and DC, in the region of the transmembrane helical bundle (spanning domain II of PLN), which is found to adopt a variety of orientations in pS16-PLN, whereas the cytoplasmic domain I is dynamically activated in the nanosecond timescale. Taken together these data indicate that in the PLN pentamer the structural fluctuations of the cytoplasmic and transmembrane regions are coupled under different timescales, an observation that could not be attained in previous structural studies^29^,^32^,^43^,^44^. This result may hold fundamental biological insights into the mechanisms of regulation of calcium uptake in the sarcoplasmic reticulum. Indeed, the coupling between cytoplasmic and transmembrane regions has been shown to be critical in the overall regulation process of SERCA^45^. The observed enhancement in structural dynamics upon phosphorylation may be at the origin of the perturbation in the equilibrium between PLN monomers and pentamers, which is key to regulate the amount of free monomers available for SERCA binding and therefore the apparent affinity of SERCA for Ca^2+^ in its physiological window^29^,^32^,^46^,^47^.

It was previously postulated that calcium uptake might possibly occur via the direct channelling through an interstitial pore at the quaternary interface of the pentameric PLN. This model has been supported by biophysical and electrochemical measurements^46^, but dismissed by structural characterisations and subsequent electrochemical and theoretical studies, showing that the interfacial pore is too narrow and hydrophobic to enable an energetically favourable transport of calcium ions^29^,^32^,^34^,^48^. We investigated the possibility that calcium transport through the pore could be achieved via structural fluctuations of the pentamer, with particular focus on the phosphorylated state. These fluctuations could generate transient structures featuring a partially distorted and more open interfacial pore thereby lowering effectively the energy barrier of Ca^2+^ channelling. Indeed, protein dynamics have been repeatedly shown to favour structurally unfavourable processes, such as catalysis or product release in enzymatic reactions, by producing transient distortions of the protein fold^49^,^50^,^51^. The present structural ensembles, however, dismiss the possibility of Ca^2+^ channelling via the distortions of the interfacial pore. Indeed, even the extreme conformations at the boundaries of the major collective twisting motion of the transmembrane helical bundle (Fig. 5) do not alter the local topology in such a way to create a transient opening of the hydrophobic pore. The characteristics of the pore, as well as the internal motions of domain II helices, are unaltered upon phosphorylation despite the huge differences in the overall topological distribution of the helical bundle (Fig. 3a)

In conclusion, our investigation identifies an approach to accurately refine structures and dynamics of oligomeric MPs and underlines the importance of conformational ensembles to describe these highly dynamical and structurally heterogeneous protein states. This framework provides a new and powerful tool to characterise the underlying mechanisms governing the biological function of these fundamental biomacromolecules under physiologically relevant conditions, as here shown with the accurate description of the dynamical activation of the PLN pentamer upon phosphorylation.

## Methods

### CSA and DC mathematical models for restrained MD simulations

CSA and DC values for the amide nitrogen are back-calculated from the three dimensional coordinates of the protein backbone atoms as previously described^42^. In general terms, CSA can be described by a 3 × 3 tensor centered on the amide nitrogen, with the following expression:


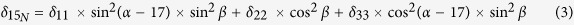


where δ_11_, δ_22_ and δ_33_ are respectively set to 64.0, 76.0, 216.9 ppm for non-glycine residues and 46.5, 66.3, 211.6 ppm for glycine. Since PLN contains no glycine residues, we only employed the first set of values. α and β are the Euler angles (in degrees) used to transform from the laboratory frame to the principal axis frame.

The DC is dependent on the length of the amide N-H bond and its angle with respect to the external magnetic field, θ:


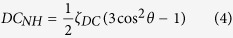


where the ζ_DC_ constant is set to 10.52 kHz.

At every step of the simulation, CSA and DC are back-calculated and compared to the corresponding experimental values. An experimental term (eq. 5) is therefore added to the force field for molecular dynamics simulations


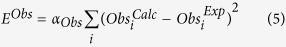


where α_ACS,DC_ is the harmonic constant and *Obs* is a generic term for any experimental observable (CSA and DC in our case). We implemented a flat-bottom harmonic potential to account for the experimental error. As a result, no force is applied if the disagreement between the data and the back-calculated values is within experimental error. In the absence of experimental errors in the dataset, we used default errors of ±5 ppm for CSA and ±0.5 kHz for DC. Restraining potentials were computed under the ensemble-averaged protocol (see below), which fulfills the maximum entropy principle^52^,^53^,^54^. In all restrained simulations, the values of the constants increased from zero to the maximum value (Table 1) during an equilibration phase of 20 ns, followed by a sampling phase of variable length. The CSA and DC restraints were implemented by modifying the GROMACS package^55^.

### Replica and internal averaging

Ensemble-averaged restrained simulations account for the averaging of experimental observables within the ensemble of conformations that compose the sample^26^. To account for this property, in replica-averaged restrained MD multiple copies (known as replicas) of the system are simulated simultaneously and independently. At every step, the NMR observables are back-calculated for each replica and averaged amongst the *M* replicas:


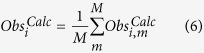


This scheme enables the restraining of the NMR data at an ensemble level. In addition to the ensemble averaging, homo-oligomeric proteins are formed by multiple copies of the same molecule, which further contribute to the averaging of the NMR data. To account for this property, we implemented internal averaging that occurs in homo-oligomeric proteins amongst the P protomers that make up the quaternary structure and combined this with the replica averaging scheme:


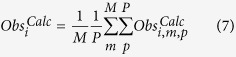


## Additional Information

**How to cite this article**: Sanz-Hernández, M. *et al.* Accurate Determination of Conformational Transitions in Oligomeric Membrane Proteins. *Sci. Rep.* **6**, 23063; doi: 10.1038/srep23063 (2016).

## Supplementary Material

Supplementary Information

Supplementary Video 1

## Figures and Tables

**Table 1:** Force constant values for the different simulation setups.

No. of replicas	Replica averaging		Replica & internal averaging	
	α_CSA_(J/(mol × ppm^2^))	α_DC_ (J/(mol × kHz^2^))	α_CSA_(J/(mol × ppm^2^))	α_DC_ (J/(mol × kHz^2^))
1	4.5	150	12.5	250
2	12.0	200	12.5	250
3	12.5	350	12.5	350
4	12.5	375	12.5	375
8	20.0	800	20.0	800
16	25.0	1000	25.0	1000
