# Supplementary material for: Accurate Determination of Conformational Transitions in Oligomeric Membrane Proteins
Source: Sci Rep. 2016 Mar 15;6:23063. doi: 10.1038/srep23063 (PMC4791661; doi:10.1038/srep23063)
Supplement: Supplementary Information [file srep23063-s1.pdf]

Supplementary Material

Accurate Determination of Conformational Transitions in Oligomeric Membrane Proteins.

Máximo Sanz-Hernández[1], Vitaly Vostrikov[2], Gianluigi Veglia[2] and Alfonso De Simone[1]

[1]*Department of Life Sciences, Imperial College London, South Kensington, London, SW7 2AZ, UK.*
[2]*Department of Chemistry & Department of Biochemistry, Molecular Biology & Biophysics, University of Minnesota, 6-155 Jackson Hall 321 Church st. SE, Minneapolis, MN 55455, USA*

Supplementary material

Supplementary Methods

MD simulation setup of phospholamban

The PLN pentamer consists of five copies of a 52 residue chain, with a serine residue at position 16 that can be phosphorylated. Starting structures were obtained in previous works with a hybrid solution NMR and oriented ssNMR approach[1,2] (PDB codes: 2KYV and 2M3B for non-phosphorylated and pS16 respectively). Both forms were embedded in lipid bilayers that reproduced the 4:1 DOPC to DOPE ratio of the SR membrane, employed in the oriented ssNMR experiments, and solvated with Tip3p water models[3]. Lipids were modelled using the all-atom generalized AMBER force field[4]. The non-phosphorylated pentamer was embedded in a membrane of 215 DOPC and 54 DOPE molecules, and solvated with 10,731 water molecules, totalling to 73,284 atoms. The pS16-PLN was simulated in a membrane of 291 DOPC and 73 DOPE lipids, solvated with 12,716 waters for a total of 92,193 atoms. Both systems were simulated under two different protein force fields: AMBER99SB-ILDN[5] and CHARMM36[6].

The solvated protein/bilayer systems were equilibrated during 100 ns of MD at 300K restraining the position of heavy atoms with a force constant of 1,000 kJ/(mol × nm^2), followed by 50 ns of unrestrained MD. All simulations were carried out in the NPT ensemble. Temperature was coupled with the V-rescale method[7], using a coupling constant of 0.1 ps. Pressure coupling was performed with the semi-isotropic Berendsen method[8], with a coupling constant of 1 ps and a reference pressure of 1 bar. All simulations were performed under periodic boundary conditions, with an integration step of 2 fs and using LINCS algorithm for constraints[9]. The particle-mesh Ewald method[10] was used to account for electrostatic interactions.

Calibration of the method using the reference ensemble calculations

To calibrate the optimal number of replicas, the use of internal averaging and force constants for the restraints, we here adopted the reference ensemble approach[11,12]. Briefly, an ensemble of conformations of the PLN pentamer embedded in a lipid bilayer was generated by using unrestrained MD simulations (100 ns) using using the AMBER99SB-ILDN force field. From the structures of this *reference ensemble*, CSA and DC data were back-calculated and employed as restraints in simulations using the CHARMM36 force field. In the absence of NMR restraints, CHARMM36 would sample a conformational phase space having different characteristics than that explored by AMBER99SB-ILDN. By comparing the restrained CHARMM36 ensemble and the *reference ensemble* (AMBER99SB-ILDN), which generated the set of synthetic CSA and DC used to restrain CHARMM36, we could carry the calibration of the optimal parameters. This *in silico* experiment provides a highly accurate tool for benchmarking the quality of the method as the *restrained* and *reference* ensembles can be compared accurately using their atomic coordinates[11,12]. In particular, to compare these ensembles we focused on key parameters such as the tilt angles of transmembrane ($\boldsymbol{\tau}$) and cytoplasmic ($\boldsymbol{\vartheta}$) helices, global tilt angle of the pentamer ($\boldsymbol{\gamma}$) and distribution of intramolecular distances between C_α atoms (Fig. S1). The latter is a sensitive descriptor of atomic structures and fluctuations in protein ensembles whereas the employed tilt angles provide relevant topological factors for MPs. Using these metrics, we tested different ensemble-averaging schemes, including different numbers of replicas (M = 1, 2, 3, 4, 8 and 16) and internal averaging scheme. Evolutions of Q factors in these simulations are shown in Fig. S8. The results clearly show that the use of internal averaging improves the quality of the restrained ensembles without leading to over-fitting of the experimental data[13] (Fig. S1b). The estimation of the quality of the restrained simulations using these 12 setups suggests that optimal sampling scheme requires the combination of both internal and replica averaging, with the latter using 16 replicas. This setup generated a restrained CHARMM36 ensemble matching the conformations of the reference AMBER99SB-ILDN sampling at the level of the statistical error limit in the majority of the structural parameters considered. This result provides an extraordinary support for the accuracy of the CSA/DC restrained MD in refining structure, dynamics and topology of oligomeric MPs.

Comparison of distributions of structural parameters

In each of the generated ensembles we selected 10,000 equally spaced structures and calculated their distributions of structural and topological properties. The properties considered in the calibration were $\boldsymbol{\tau}$, $\boldsymbol{\gamma}$ and $\boldsymbol{\vartheta}$ angles, and intramolecular distances (***d***). Tilt angles refer to the angle formed between the helix vector and the normal of the membrane plane (the direction of the external magnetic field in the ssNMR experiment) (Fig. 2a).

In order to compare any two distributions of geometrical parameters, we can define a metric, D_{iff}, that computes the difference between the two distributions[11]. Let *A* and *B* be two normalized distributions. Then,

(S1) $$D_{iff} = \sum_i \left| P_i^A - P_i^B \right|$$

where *i* runs over equally spaced bins. The value of D_{iff} ranges from 0 (for identical distributions) to 2 (for completely different, non-overlapping distributions). Our comparisons were performed over 20 bins.

In the case of intramolecular distances, the distance between every pair of C_α atoms within the PLN protomer was computed for every structure of each ensemble. In a protein with *n* residues, this would yield an *n×n* matrix where each cell corresponds to one distribution of distances. When comparing two ensembles, every distribution can be compared to its equivalent generating a D_{iff} value. If done for every C_α pair, we can obtain the average D_{iff} value of these distributions as a measure of the overall similarity of the ensembles.

For each of the outlined properties we compared the unrestrained and the reference ensembles, thus generating an unrestrained D_{iff} value, D_{Unr}. In each of our synthetic setups (restrained with data from the reference ensemble) we compared the resulting ensembles to the reference, obtaining another D_{iff} value, or D_{Ens}. By comparing D_{Ens} to D_{Unr}, one can assess if the introduction of restraints improved or worsened the description of the synthetic, reference ensemble. We can define ΔD_{iff} as an absolute measure of the improvement:

(S2) $$\Delta D_{iff} = D_{Ens} - D_{Unr}$$

If the D_{iff} value of an ensemble is lower than the unrestrained ($\Delta D_{iff} < 0$), the restrained ensemble is closer to the reference ensemble and therefore provides a better description of the structural property under study.

In addition, we ran a further 50ns of sampling with the same force-field that had generated the reference. In this way, we obtained a new ensemble that is significantly similar to the reference, at the level of statistical error. The ΔD_{iff} for this ensemble therefore represents the desirable level of improvement that can be achieved by restrained sampling. The details for all setups are reported in Fig. S1.

Restrained simulations for the synthetic ensemble test were performed for 50ns per replica per setup, with an initial 20ns of force equilibration followed by 30ns of sampling. Unrestrained simulations and ensembles restrained with the experimental data were sampled for 100 ns per replica. For each of these simulations, the 20ns phase of force equilibration was followed by 80ns of sampling per replica, generating ensembles of 12,800 equally spaced structures (taken every 100 ps). The collective sampling time of the simulations presented in this study amounts to 10 µs.

Calculation of NMR, Structural and topological parameters from the ensembles.

NMR order parameters S^2 for amide N-H bond vectors were back-calculated by superimposing the structures of the ensemble with the least-square fitting algorithm (using the C_α atoms of the TM segments of PLN) and by adopting the approach described by Best et al[14]. In the case of the S^2 of the global axis of the TM helical bundle (Fig. 4e), the axis of the TM bundle was considered instead of the amide N-H bond vectors.

Tilt angles "τ"(Fig. 2a) were computed between the membrane normal ($\mathbf{B_0}$) the axis of the TM helices ($\mathbf{h_{TM}}$, in Fig. 2). Each helical axis was calculated by linear least-squares fitting of the backbone atoms in the helix. The TM helices were defined as residues 25 to 51, and cytoplasmic helices (θ angle) as residues 3 to 14.

Average tilt angles "γ" (Fig. 2b) of the TM helical bundle were computed between the membrane normal ($\mathbf{B_0}$) and the axis of the TM helical bundle ($\mathbf{h_{bundle}}$). The latter is the normalised sum of the axes of each individual TM helix of the bundle.

Pore radius were calculated by using the program MOLE 2.0[15] and by aligning all the structures of the ensemble using the backbone atoms of the TM segments for the least-squares fitting.

Q-factors

The agreement between back-calculated and experimental NMR data is reported in terms of Q-factors, which are calculated according to the following expression:

$$\text{(S3)} \qquad Q = \sqrt{\frac{\sum_i \left(Obs_i^{Exp} - Obs_i^{Calc}\right)^2}{\sum_i \left(Obs_i^{Exp}\right)^2}}$$

References

1. Vostrikov, V. V., Mote, K. R., Verardi, R. & Veglia, G. Structural dynamics and topology of phosphorylated phospholamban homopentamer reveal its role in the regulation of calcium transport. *Structure* **21,** 2119–2130 (2013).
2. Verardi, R., Shi, L., Traaseth, N. J., Walsh, N. & Veglia, G. Structural topology of phospholamban pentamer in lipid bilayers by a hybrid solution and solid-state NMR method. (Supl). *Proc. Natl. Acad. Sci. U. S. A.* **108,** 9101–9106 (2011).
3. Jorgensen, W. L., Chandrasekhar, J., Madura, J. D., Impey, R. W. & Klein, M. L. Comparison of simple potential functions for simulating liquid water. *J. Chem. Phys.* **79,** 926 (1983).
4. Dickson, C. J., Rosso, L., Betz, R. M., Walker, R. C. & Gould, I. R. GAFFlipid: a General Amber Force Field for the accurate molecular dynamics simulation of phospholipid. *Soft Matter* **8,** 9617 (2012).
5. Lindorff-Larsen, K. *et al.* Improved side-chain torsion potentials for the Amber ff99SB protein force field. *Proteins* **78,** 1950–1958 (2010).
6. Best, R. B. *et al.* Optimization of the additive CHARMM all-atom protein force field targeting improved sampling of the backbone phi, psi and side-chain chi-1 and chi-2 Dihedral Angles. *J. Chem. Theory Comput.* **8,** 3257–3273 (2012).
7. Bussi, G., Donadio, D. & Parrinello, M. Canonical sampling through velocity rescaling. *J. Chem. Phys.* **126,** (2007).
8. Berendsen, H. J. C., Postma, J. P. M., van Gunsteren, W. F., DiNola, a & Haak, J. R. Molecular dynamics with coupling to an external bath. *J. Chem. Phys.* **81,** 3684–3690 (1984).
9. Hess, B., Bekker, H., Berendsen, H. J. C. & Fraaije, J. G. E. M. LINCS: A linear constraint solver for molecular simulations. *J. Comput. Chem.* **18,** 1463–1472 (1997).
10. Darden, T., York, D. & Pedersen, L. Particle mesh Ewald: An N log(N) method for Ewald sums in large systems. *J. Chem. Phys.* **98,** 10089 (1993).
11. De Simone, A., Richter, B., Salvatella, X. & Vendruscolo, M. Toward an accurate determination of free energy landscapes in solution states of proteins. *J. Am. Chem. Soc.* **131,** 3810–1 (2009).
12. De Simone, A., Montalvao, R. W. & Vendruscolo, M. Determination of Conformational Equilibria in Proteins Using Residual Dipolar Couplings. *J. Chem. Theory Comput.* **7,** 4189–4195 (2011).
13. Richter, B., Gsponer, J., Várnai, P., Salvatella, X. & Vendruscolo, M. The MUMO (minimal under-restraining minimal over-restraining) method for the determination of native state ensembles of proteins. *J. Biomol. NMR* **37,** 117–35 (2007).

14. Best, R. B., Clarke, J. & Karplus, M. What contributions to protein side-chain dynamics are probed by NMR experiments? A molecular dynamics simulation analysis. *J. Mol. Biol.* **349,** 185–203 (2005).
15. Sehnal, D. *et al.* MOLE 2.0: Advanced approach for analysis of biomacromolecular channels. *J. Cheminform.* **5,** 1–13 (2013).
16. Buffy, J. J. *et al.* Two-dimensional solid-state NMR reveals two topologies of sarcolipin in oriented lipid bilayers. *Biochemistry* **45,** 10939–10946 (2006).

Supplementary figures

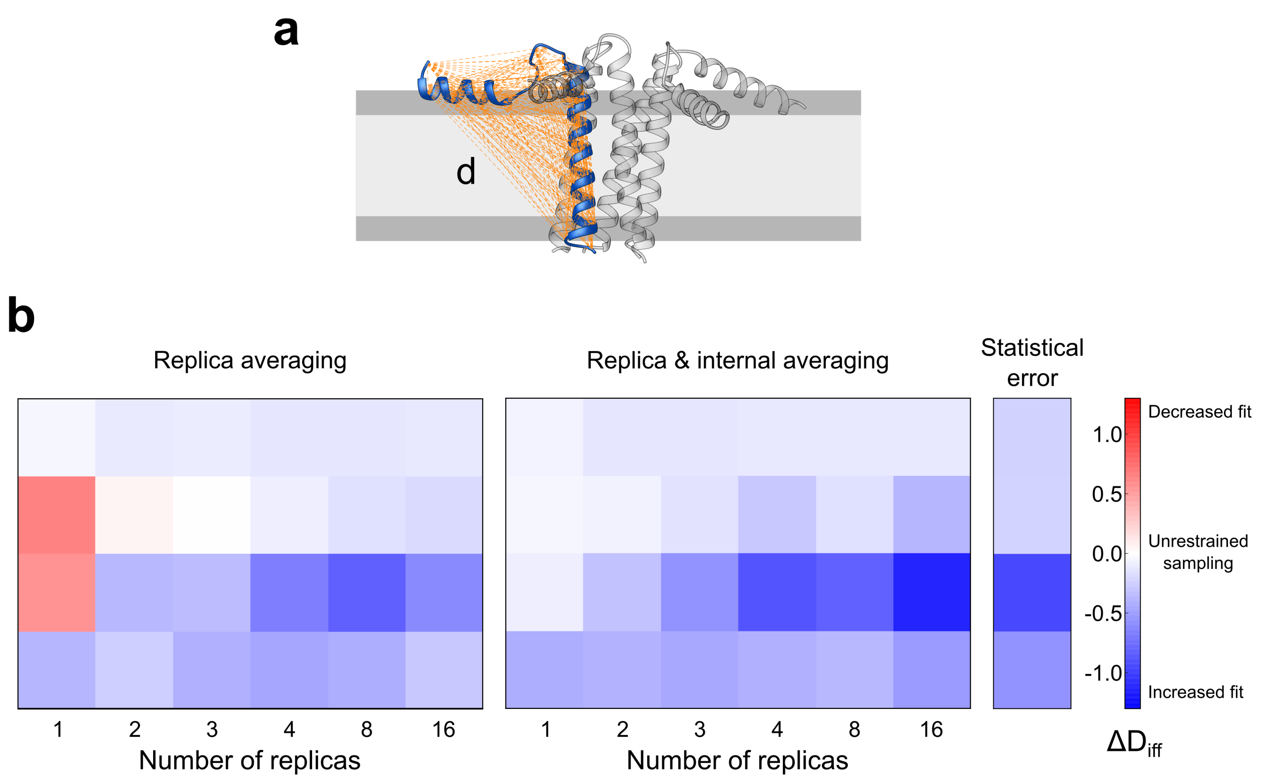

Supplementary Figure S1. Validation of the restraining protocol by means of the synthetic ensemble test. *(a) Distribution of intramolecular distances, d, between every pair of C_α atoms in each protomer. (b) For the four structural properties listed (see Fig. 2 in main text for definitions of τ, γ and ϑ), a D_{iff} value between the unrestrained ensemble and the reference ensemble was computed, which measures their difference (see Suppl. Methods). For each restrained ensemble, we calculated their corresponding D_{iff} values with respect to the reference and compared them to the unrestrained D_{iff}, obtaining the measure of improvement ΔD_{iff}. A positive ΔD_{iff} (red) indicates less similarity to the reference ensemble upon introducing the restraints. A negative ΔD_{iff} (blue) corresponds to an improvement in the overlap of the distributions, and thus a better representation of the structural property of interest. ΔD_{iff} values are reported for the 12 setups tested. The rightmost column corresponds to a 50ns unrestrained ensemble generated with the same force-field as the reference, and represents the level of statistical error in the sampling.*

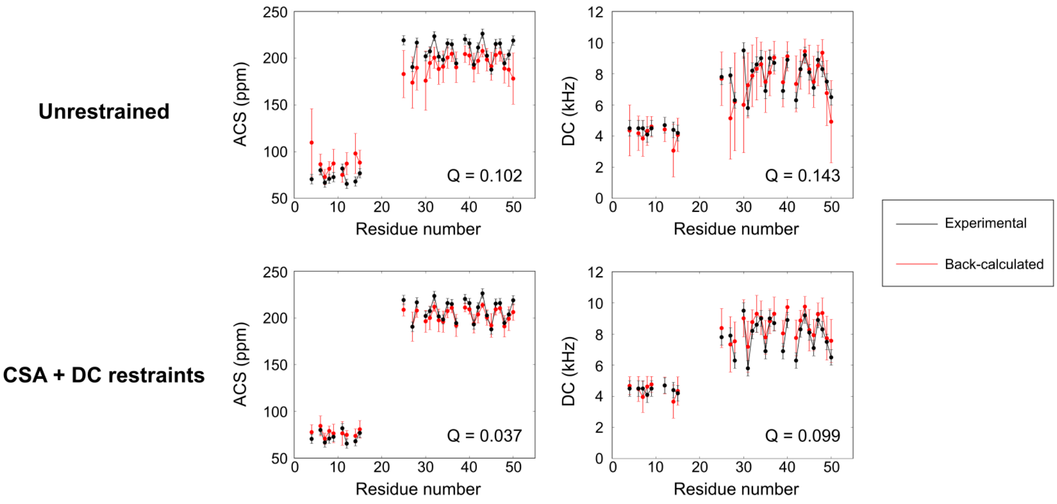

***Supplementary Figure S2. Agreement between simulations and experimental data of non-phosphorylated* PLN.** *The introduction of the restraints improves the agreement with the experimental data in all our samplings. Overall agreement is expressed in terms of Q-factors (see Supplementary Methods). This figure shows the results from the non-phosphorylated AMBER ensembles.*

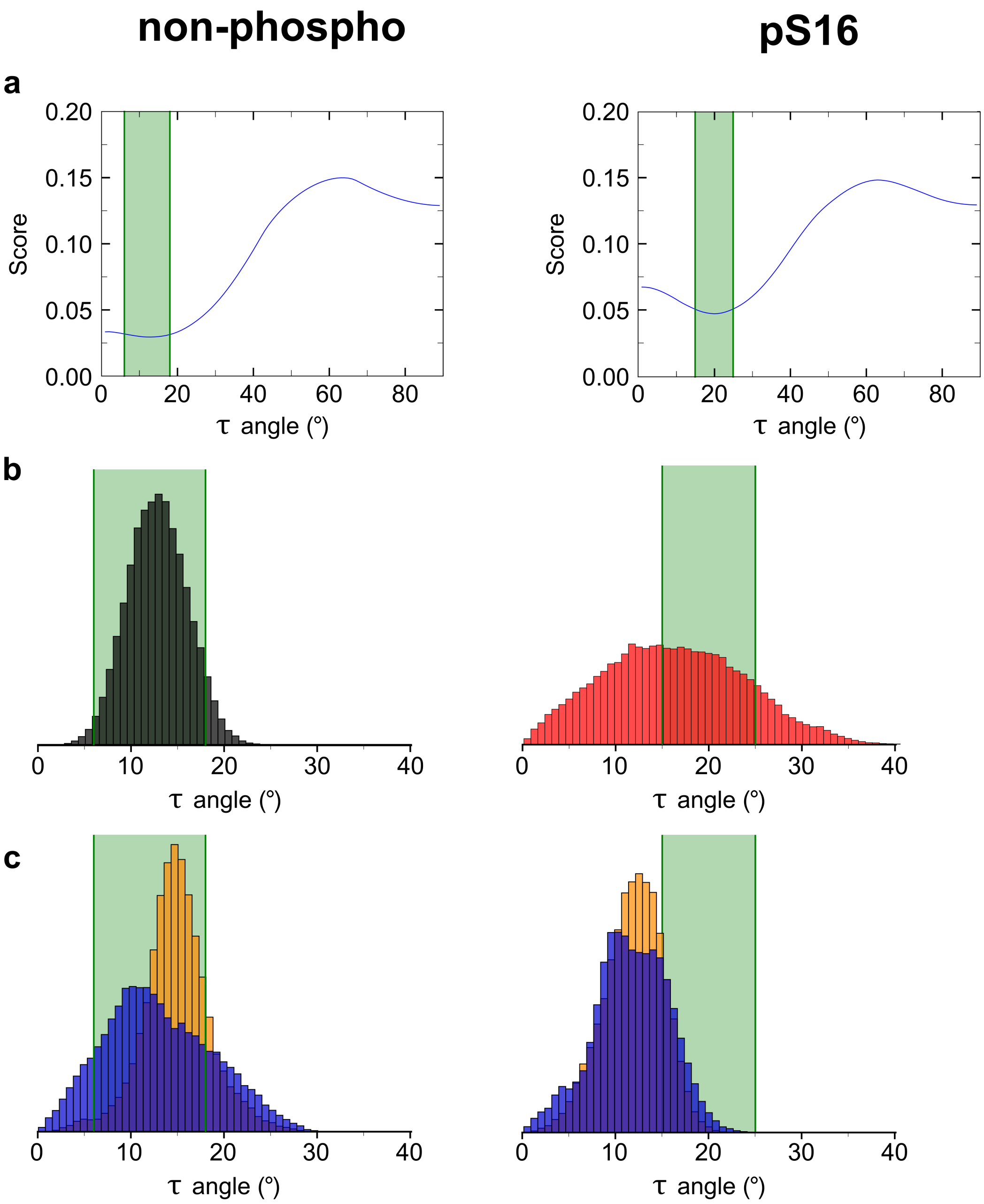

Supplementary Figure S3. Validation of the τ angles in the restrained CHARMM36 simulations and by using the analytical approach[16]. *(a) Score of the static fitting method, where lower scores correspond to better fits. The areas with the best scores are highlighted in green. (b) Distributions of τ angles back-calculated from the restrained ensembles, with the static best-fit area superposed in green. (c) τ angle distributions back-calculated from the unrestrained ensembles.*

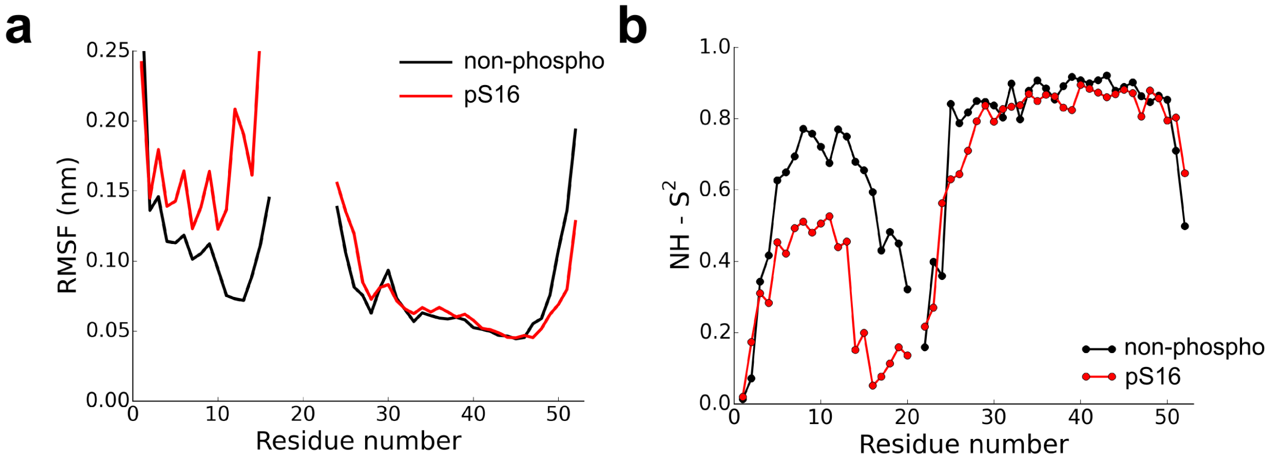

Supplementary Figure S4. Effect of phosphorylation in the AMBER restrained ensembles. *(a) RMSF of the C_α atoms of PLN. The calculation was performed separately for the two domains of the protein, in order to remove biasing effects of global, inter-domain motions. (b) Back-calculated S^2 parameters of the backbone amide N-H bonds.*

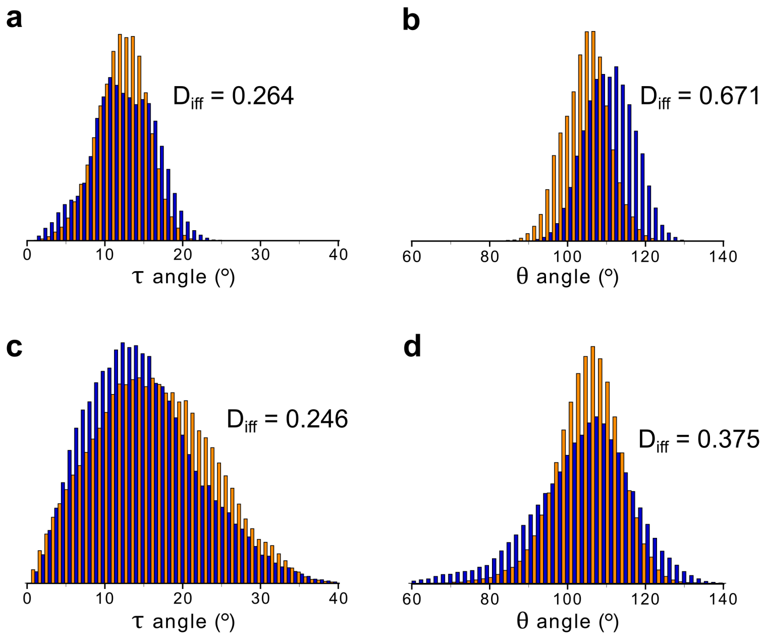

Supplementary Figure S5. Convergence of the restrained simulations of pS16-PLN, pentamer. *Distributions of τ and ϑ angles in unrestrained (a,b) and restrained (c,d) simulations. CHARMM samplings are shown in orange, and AMBER in blue. The difference between every pair of distributions is reported as a D_{iff} value (see Supplementary Methods).*

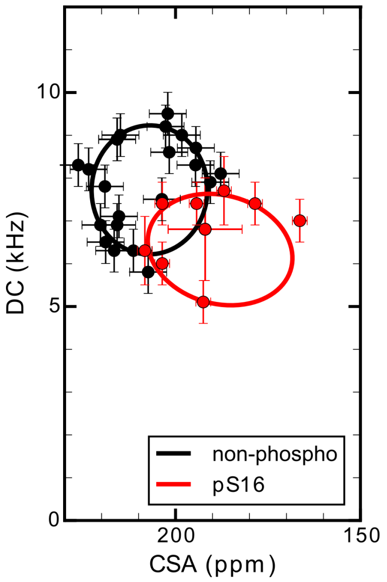

Supplementary Figure S6. PISEMA representation of the experimental data. *Data for the TM segment of non-phosphorylated and pS16 pentameric PLN in mechanically aligned bilayers are shown in black and red, respectively. The observed wheel patterns are characteristic of α-helical segments.*

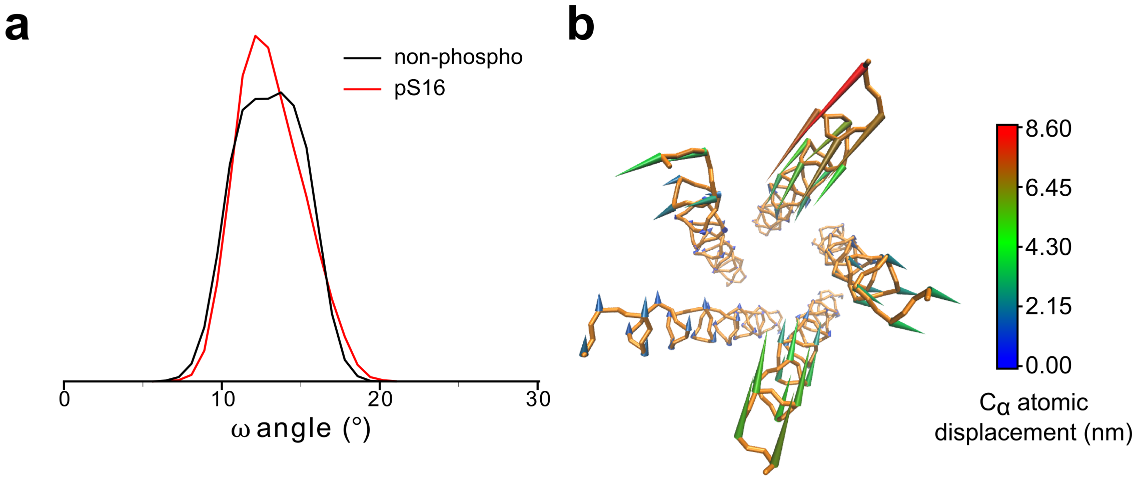

Supplementary Figure S7. Internal motions of the transmembrane segment in non-phosphorylated PLN. *(a) Distribution of ω angles in non-phosphorylated PLN and pS16 PLN. (b) Principal Component Analysis in non-phosphorylated PLN. The internal motions of the N-terminal part of the TM helices are observed in both non-phosphorylated and pS16 PLN. Cones show the motion mode of* C_α *atoms along the first eigenvector. A video showing the motions sampled by the first eigenvector is provided as supplementary material.*

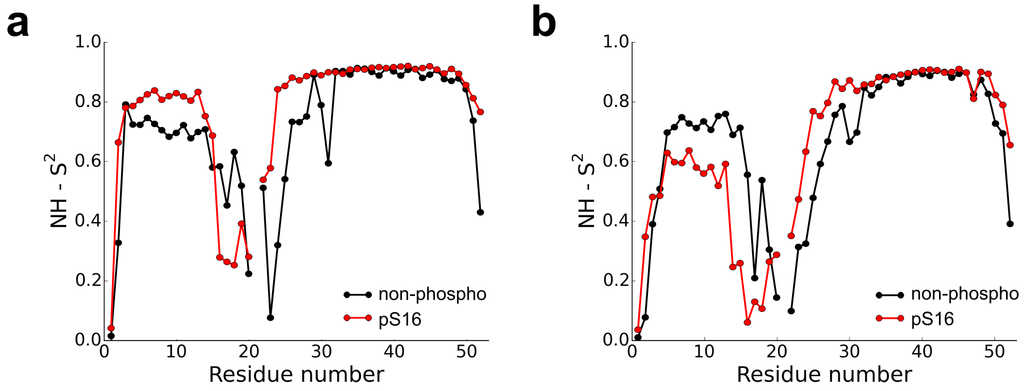

Supplementary Figure S8. Back-calculated S^2 parameters in unrestrained simulations. *Order parameters were calculated for the backbone amide N-H bond vector of each residue in PLN. Panels (a) and (b) for CHARMM and AMBER simulations, respectively.*

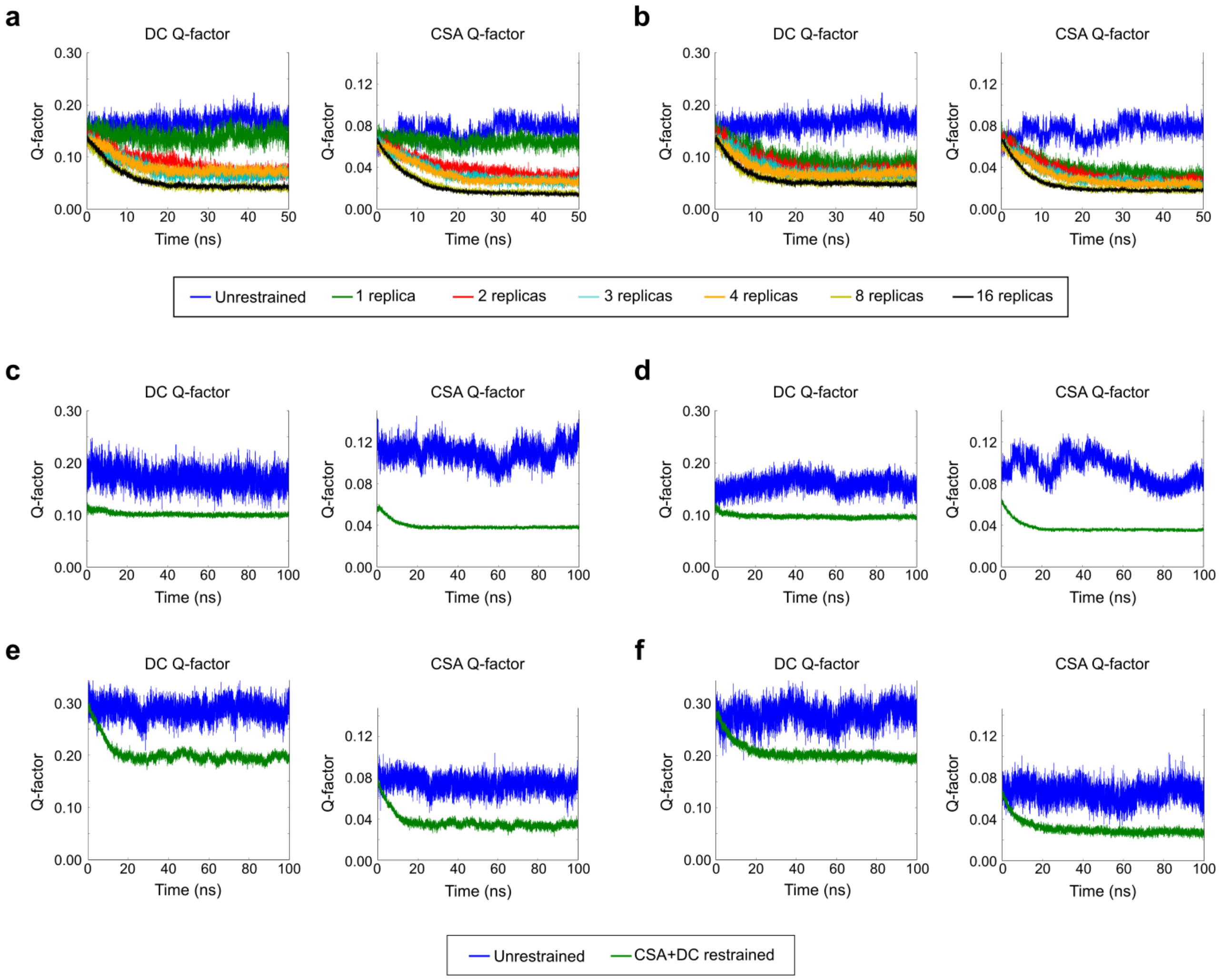

Supplementary Figure S9. Convergence in the simulations. *Q-factors are shown as a function of time for all simulations. Reference ensemble validation with replica averaging (a) and combination of replica & internal averaging (b). AMBER (c) and CHARMM (d) sampling of the non-phosphorylated pentamer. AMBER (e) and CHARMM (f) sampling of the pS16 pentamer.*
